# Effect of Welding Peak Temperature on Microstructure and Impact Toughness of Heat-Affected Zone of Q690 High Strength Bridge Steel

**DOI:** 10.3390/ma14112981

**Published:** 2021-05-31

**Authors:** Yue Zhang, Jun Xiao, Wei Liu, Aimin Zhao

**Affiliations:** Collaborative Innovation Center of Steel Technology, University of Science and Technology Beijing, Beijing 100083, China; b20180502@xs.ustb.edu.cn (Y.Z.); S20191289@xs.ustb.edu.cn (J.X.); b20160467@xs.ustb.edu.cn (W.L.)

**Keywords:** bridge steel, welding thermal simulation, peak temperature, heat-affected zone, mechanical properties

## Abstract

The effect of peak temperature (T_P_) on the microstructure and impact toughness of the welding heat-affected zone (HAZ) of Q690 high-strength bridge steel was studied using a Gleeble-3500 thermal simulation testing machine. The results show that the microstructure of the inter critical heat-affected zone (ICHAZ) was ferrite and bainite. The microstructure of fine grain heat-affected zone (FGHAZ) and coarse grain heat-affected zone (CGHAZ) was lath bainite (LB), lath martensite (LM), and granular bainite (GB), but the microstructure of FGHAZ was finer. With the increase in peak temperature, the content of LB and GB decreased, the content of LM increased, and the lath bundles of LM and LB gradually became coarser. With the increase in peak temperature, the grain size of the original austenite increased significantly, and the impact toughness decreased significantly. When the peak temperature was 800 °C, the toughness was the best. For CGHAZ, the peak temperature should be less than 1200 °C to avoid excessive growth of grain and reduction of mechanical property.

## 1. Introduction

With the development of long-span steel bridge construction, bridge structure and performance requirements are constantly improving. The traditional structural steel plate cannot fully meet the requirements of bridge design and construction [1,2]. Q690, as the representative of high-performance bridge steel, has a high strength and low yield ratio, which not only meets the requirements of high-performance bridge steel but also dramatically improves economic and environmental efficiency [3]. Q690 low-alloy high-strength steel has good strength and toughness, which is widely used in the coal industry, oil and gas drilling, ocean engineering, machinery manufacturing, and other fields [4]. With the increase of yield strength and alloy element content of steel, it is more challenging to control critical indicators, such as yield strength ratio, toughness, and weldability of low-alloy high-strength steel. The main problems of high-strength steel welding include cold crack tendency, embrittlement, and softening in the heat-affected zone [5,6]. The heat-affected zone (HAZ) has a certain gradient distribution in the microstructure and performance and becomes the weakest part in the welding joint [7]. Especially, the coarse grain HAZ (CGHAZ), which is adjacent to the weld fusion line.

Recent research has focused on the effect of heat input on the microstructure and properties of HAZ [5,8,9,10]. In general, welding heat input can be related to Δt_8/5_. Δt_8/5_ means the time required to cool from 800 °C to 500 °C. Wen et al. [11] thought that reasonable control of heat input is the key to preventing welding cold cracks by studying ultra-high-strength structural steel welding joint at different heat inputs. It was pointed out that the heat input should be higher than 7.5 kJ/cm to avoid the formation of welding cold crack and twinned martensite in CGHAZ. It is generally believed that welding stress and hardened microstructure of high strength and low alloy (HSLA) steel welded joints are the factors that promote cold cracks in welded joints. The so-called hardened microstructure mainly refers to martensite. Martensite is a supersaturated solid solution of carbon in α iron. It is a brittle microstructure. Under certain stress conditions, brittle fracture will occur, so cracks are easy to form and develop. The HAZ has undergone recrystallization and grain growth processes, both of which are affected by the transient and high-temperature characteristics of the welding process, and numerical simulation methods can reproduce this process well [12]. Bayock et al. [13] studied the effect of heat input on the mechanical properties and microstructure of dissimilar welded joints of 690 MPa steel through numerical simulation. The results show that the welding heat input should be reasonably controlled and limited, especially for thick plates, because the softening effect of welded joints is proportional to the thickness of plates. Cao et al. [14], through the study of high-strength low-alloy steel, proposed a micro reaction mechanism that the impact toughness of coarse grain heat-affected zone decreases with the increase of welding heat input. Yang et al. [7] reported on the effect of heat input on the microstructure and fracture toughness of the simulated CGHAZ of high-strength low-alloy steel. With the increase in heat input, the content of the martensite-austenite constituent (M-A constituent) increased. The island-shaped M-A constituent can prevent microcrack generation, but the coarse and massive M-A constituent can seriously reduce fracture toughness. Li et al. [15] studied the influence of M-A constituent in the inter critical heat-affected zone (ICHAZ) of low-carbon bainite steel Q690 steel on impact toughness. The results show that the brittleness of the ICHAZ zone depends on the deformability and size of the M-A constituent. When the size of the M-A constituent exceeds the critical value (2.0 µm), ICHAZ embrittles. The effect of peak temperature on the microstructure and properties of heat affected zone was also studied [16,17,18,19]. Especially the influence of peak temperature on CGHAZ. Peak temperature will change austenite grain size and precipitation behavior, which has a significant effect on the strength and toughness of the alloy [17]. Meanwhile, the austenite grain size of HAZ also has an essential influence on the microstructure and properties of the steel. Khalaj et al. [20] studied the effects of peak temperature and heating rate on grain size and proposed a model for the dissolution of NbCN precipitates to predict the austenite grain size in an Nb/Ti microalloyed linepipe steel weld HAZ. Pouraliakbar et al. [21] established a neural network model combining feed-forward topology and back propagation algorithm to predict the hardness of HAZ of X70 pipeline steel. The chemical composition and tensile test parameters were the input parameters, and the hardness was the output parameter. The experimental results coincided well with the predicted hardness from the model. Tomków et al. [22] studied the influence of temper bead welding on the hardness and structures of the heat-affected zone (HAZ) of dissimilar T-joints made under underwater conditions. The temper bead welding reduced the hardness in the HAZ of S460N and S460ML, thereby reducing the susceptibility to cold cracking. However, little attention has been paid to the effect of peak temperature on the HAZ of Q690 high-strength steel.

Therefore, this study uses a thermal simulation testing machine to conduct a welding thermal simulation test on Q690 high-strength bridge steel to study the influence of the peak temperature (Tp) on the microstructure and impact toughness of the high-strength bridge steel HAZ. This study can provide a theoretical reference for selecting and optimizing the actual welding process of 690 MPa grade high strength low-yield ratio bridge steel and the strengthening and toughening of welded joints.

## 2. Materials and Methods

### 2.1. Materials

**Figure 1:**
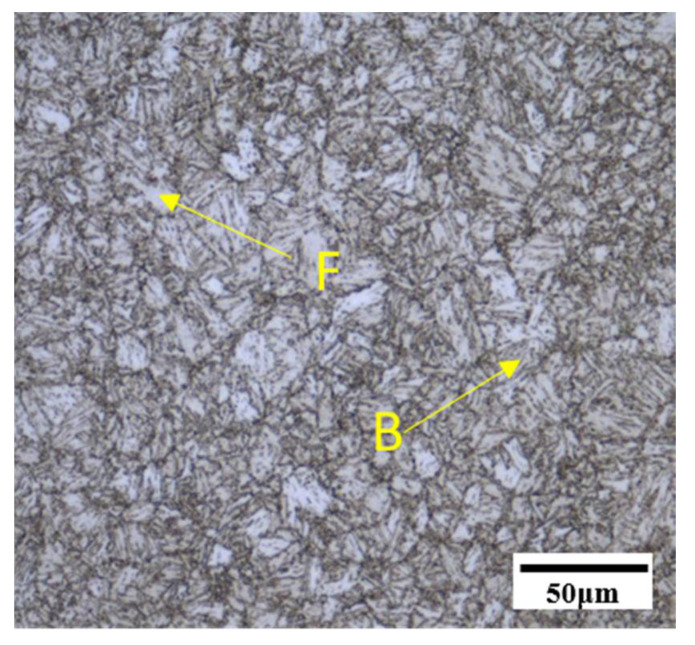
Microstructure of experimental steel.

The experimental steel was Q690 high-strength bridge steel (University of Science and Technology Beijing, Beijing, China). The chemical compositions of the experimental steel, obtained using the chemical method, are listed in Table 1, and the carbon equivalent (CE) was calculated using Equation (1). The carbon equivalent (CE) of the tested steel calculated using the carbon equivalent equation, Equation (1), was 0.66. It is generally believed that when the carbon equivalent is more than 0.6%, the hardenability of steel is strong, and cold cracks easily appear [23]. The experimental steel was melted in a vacuum induction furnace and manufactured by thermomechanical control process (TMCP) technology, critical quenching, and medium temperature tempering. The microstructure of the experimental steel was composed of ferrite and bainite, as shown in Figure 1. An alcohol solution Alcohol (Sinopharm Chemical Reagent Co., Ltd, Shanghai, China) containing 4% of nitric acid (Sinopharm Chemical Reagent Co., Ltd, Shanghai, China) (volume fraction) was used to erode the metallographic specimen. The hardness of the base metal was 304.8 HV0.5.

(1) $$\mathrm{CE}=C+\frac{\mathrm{Mn}}{6}+\frac{\mathrm{Cr}+\mathrm{Mo}+V}{5}+\frac{\mathrm{Ni}+\mathrm{Cu}}{15}$$

### 2.2. Welding Thermal Simulation Procedure

**Figure 2:**
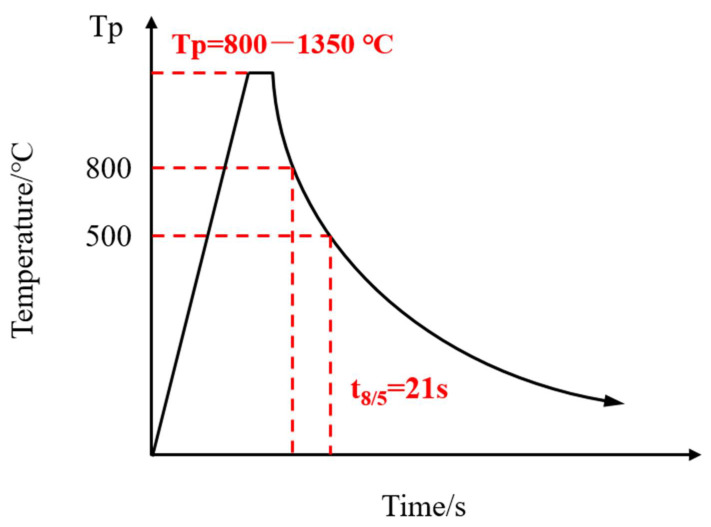
Welding thermal cycle test process with the different peak temperature.

**Figure 3:**
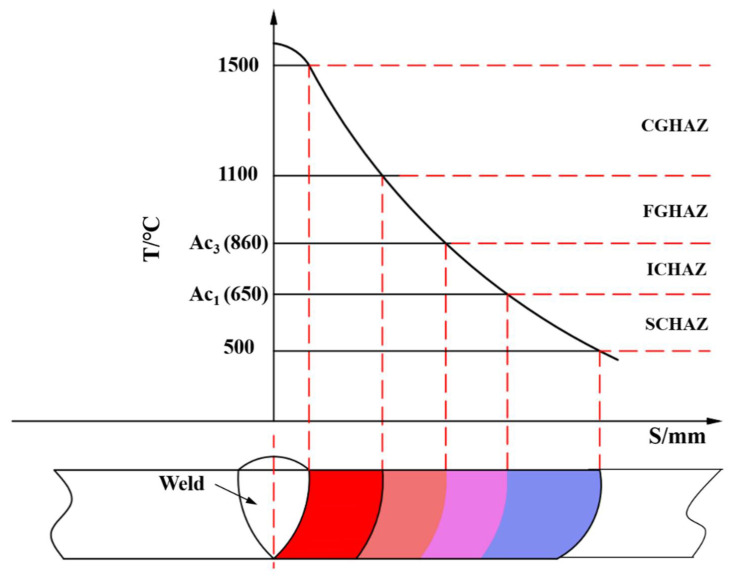
Diagram of microstructure distribution in welding heat-affected zone.

The microstructure and mechanical properties of the Q690 high strength bridge steel HAZ were simulated using a Gleeble-3500 simulation machine (Texas Dynamic Systems Inc, Austin, TX, USA). The thermal simulation specimen dimensions were 10.5 mm × 10.5 mm × 80 mm. The specimens were subjected to welding thermal cycles with 20 kJ/cm heat inputs and different peak temperatures (T_P_). Based on the correlation between heat inputs (E) and t_8/5_, the corresponding t_8/5_ for the 20 kJ/cm heat inputs was 21 s [10,17]. Simulated welding thermal cycles of the experimental steel HAZ are shown in Figure 2. The simulated samples were heated to different peak temperatures at a heating rate of 100 °C/s and then held for 1 s. The cooling time of t_8/5_ was 21 s. According to the corresponding standard (YBT 5127-1993), the temperatures of Ac1 and Ac3, measured using a dilatometer, were 650 °C and 860 °C, respectively. The peak temperature of 800 °C was selected to simulate the microstructure of ICHAZ, the peak temperatures of 900 °C and 1050 °C was selected to simulate the microstructure of the fine grain heat-affected zone (FGHAZ), and the peak temperatures of 1150 °C, 1200 °C, 1250 °C, 1300 °C, and 1350 °C were selected to simulate the microstructure of CGHAZ. Figure 3 shows a diagram of microstructure distribution in the welding heat-affected zone.

### 2.3. Microstructural Characterization

The metallographic samples were prepared according to the GB/T 13298-2015 standard. The metallographic specimens of simulated HAZs were cut on the steel plate and then ground polished and etched with 4% nitric acid alcohol. The microstructures of the samples were observed using an optical microscope (OLYMPUS LEXT OLS4000 laser confocal microscope, Olympus corporation, Tokyo, Japan) and a QUANTA FEG-450 field emission environmental scanning electron microscope (SEM, FEI, Hillsboro, OR, USA). The crystallographic characteristics of the samples were investigated via electron backscatter diffraction (EBSD, Oxford Instruments group, Britain). The EBSD sample preparation used the following steps; wire cutting, oil removal, sample mosaic, grinding, and polishing (mechanical polishing and electrolytic polishing). Electrolytic polishing used 10% perchloric acid (Sinopharm Chemical Reagent Co., Ltd Shanghai, China) + 90% alcohol. The voltage, electric current, and time of polishing were 15 V, 0.5 A and, 5–10 s, respectively. The EBSD data processing and analyses were carried out using HKL Channel 5 software (Oxford Instruments, Britain).

The transmission electron microscopy (TEM, JEM-2100, JEOL, Tokyo, Japan) samples were mechanically ground to 40–50 μm in thickness and then were made by punching disks with a 3-mm diameter. Then, twin-jet electropolishing was performed using a 5 vol% perchloric acid and alcohol at a voltage of 40 V.

### 2.4. Mechanical Properties

The microhardness of the experimental steel was detected using a THV-1MD type Vickers hardness tester (TEST-TECH Co., Ltd, Shanghai, China) with a load of 1000 g. The experimental process was carried out strictly in accordance with GB/T 4340.1-2009. The hardness value was the average of ten hardness values.

The impact test was carried out in accordance with the GB/T 229-2007 standard. Charpy impact tests were performed with standard specimens at −40 °C. The size of the sample was 10 mm × 10 mm × 55 mm. The Charpy V-notch was located in the section of the thermocouple. The V-notch has an angle of 45° and a depth of 2 mm. The specimen was subjected to a JBDW-300dD swing impact test machine (JiNan Gaosheng R & D center and production base, Shangdong, China). The value is the average of three sample values.

## 3. Results and Discussions

### 3.1. Microstructure

**Figure 4:**
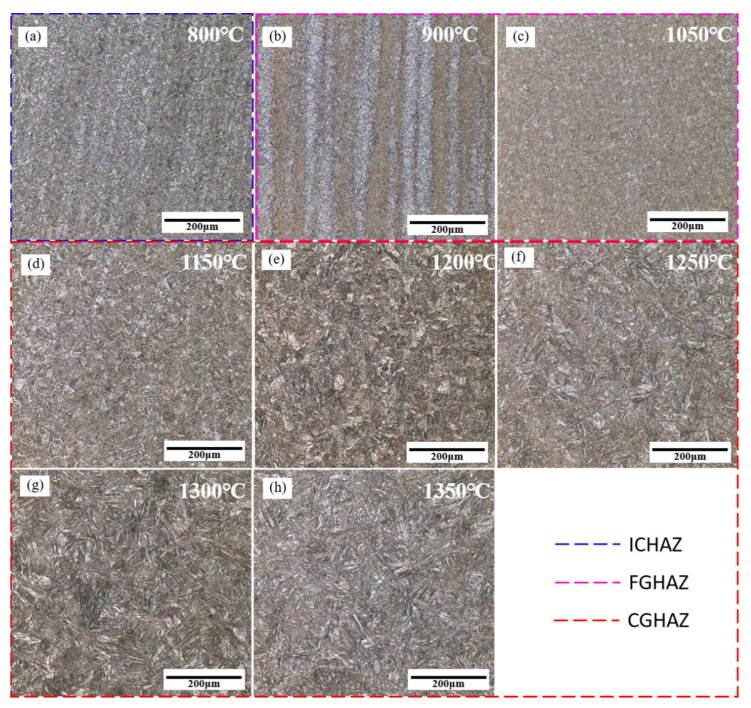
Microstructure of simulated HAZ at different peak temperatures. (**a**) 800 °C, (**b**) 900 °C, (**c**) 1050 °C, (**d**) 1150 °C, (**e**) 1200 °C, (**f**) 1250 °C, (**g**) 1300 °C, and (**h**) 1350 °C.

**Figure 5:**
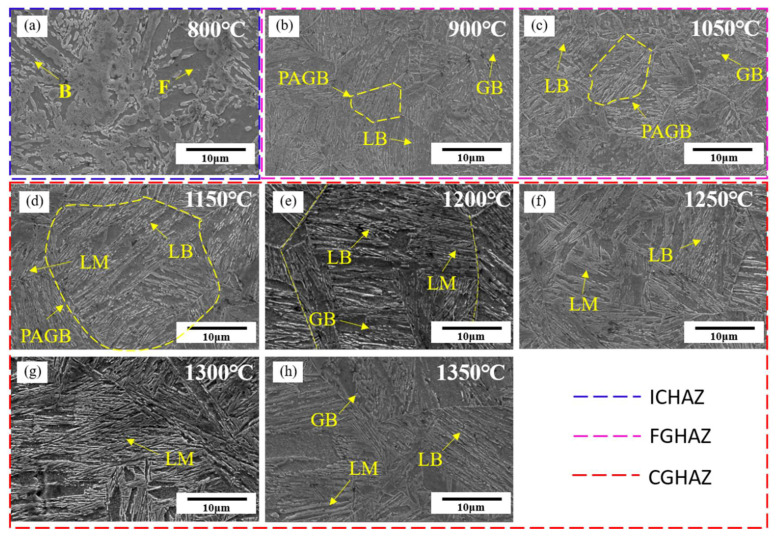
SEM microstructure of simulated HAZ at different peak temperatures. (**a**) 800 °C, (**b**) 900 °C, (**c**) 1050 °C, (**d**) 1150 °C, (**e**) 1200 °C, (**f**) 1250 °C, (**g**) 1300 °C, and (**h**) 1350 °C.

The microstructure of the simulated specimens at different peak temperatures was observed and are shown in Figure 4 and Figure 5. When the peak temperature was 800 °C, the microstructures of simulated ICHAZ were composed of bainite (B) and ferrite (F), and M-A constituent could be observed between the ferrite lathes (Figure 4a and Figure 5a). Since 800 °C is between the temperatures of Ac1 and Ac3, the original microstructure of the base metal (BM) was partially austenitized, and the austenitized part was cooled to form a finer bainite microstructure. The non-austenite part underwent recovery and recrystallization, and ferrite was formed after cooling.

When the peak temperature increased to 900 °C and 1050 °C, the microstructure of FGHAZ was mainly LB, and there was also a small amount of GB and LM (Figure 4b,c and Figure 5b,c). Bainite and martensite lath bundles were smaller, GB distribution was relatively dispersed, prior austenite grain boundary (PAGB) was more clearly visible, and the grain size was smaller. The microstructure of FGHAZ at 900 °C was smaller than that at 1050 °C.

When the peak temperature increased to 1150 °C, 1200 °C, 1250 °C, 1300 °C, and 1350 °C, the dominant microstructures of simulated CGHAZ was LM, and a small amount of GB and LB were also available (Figure 4d–h and Figure 5d–h). When the peak temperature was 1150 °C, the lath bundles of martensite and bainite were relatively fine, and the PAGB is clearly visible, and the grains were relatively fine. When the peak temperature exceeded 1200 °C, a complete prior austenite grain could not be observed in the field of view (Figure 5e). With the increase in peak temperature, the content of LB and GB gradually decreased, while the content of LM gradually increased. With the increase in peak temperature, LM and LB lath bundles gradually widened, prior austenite grains grew obviously, and the microstructure of CGHAZ grew obviously.

**Figure 6:**
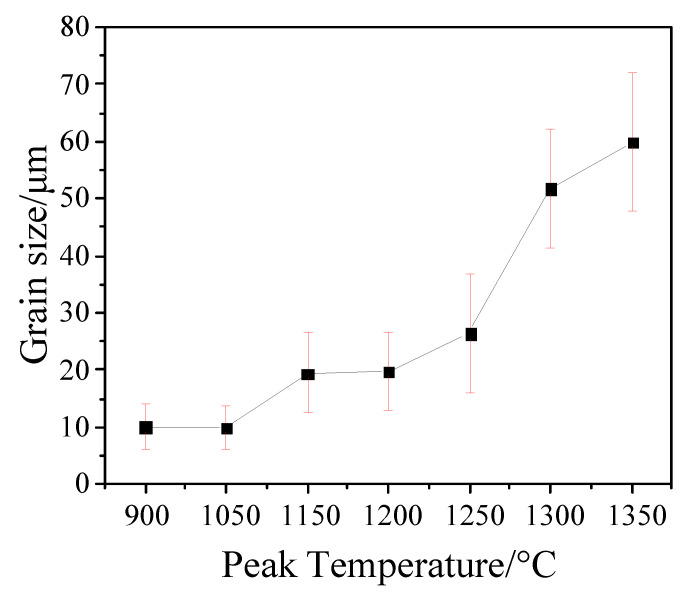
The change of original austenite grain size at different peak temperatures.

The original austenite grain size was counted using ImageJ software. The statistical results of the original austenite grain size at different peak temperatures are shown in Figure 6. With the increase in peak temperature, the original austenite grain size increased significantly. When the peak temperature was 900 °C, the original austenite grain size was 10.13 μm. When the temperature increased to 1050 °C, the grain size of the original austenite did not increase significantly. When the temperature increased to 1150 °C and 1200 °C, the grain size of the original austenite increased to about 20 μm. With the increase in the peak temperature, the original austenite grain size continued to increase. When the peak temperature was 1350 °C, the original austenite grain size reached the maximum (59.99 μm).

**Figure 7:**
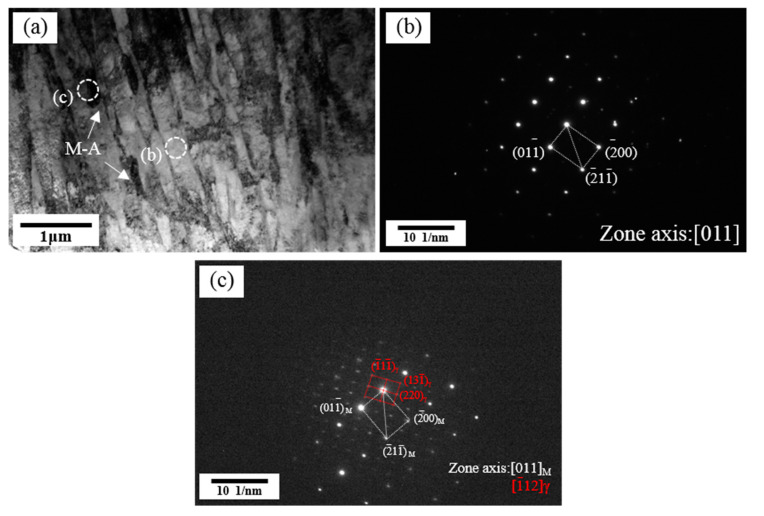
TEM micrographs of microstructure in the simulated CGHAZ at a peak temperature of 1350 °C: (**a**) microstructure, (**b**) corresponding SADP for martensite, (**c**) corresponding SADP for M-A constituent.

Figure 7 shows the TEM morphologies of the simulated CGHAZ at a peak temperature of 1350 °C. It can be seen from the figure that there are rod-like M-A constituents between martensite laths with the same orientation. The M-A constituents are detrimental to toughness. The corresponding selected area diffraction pattern (SADP) of martensite and the M-A constituent are presented in Figure 7b,c.

### 3.2. Microhardness

**Figure 8:**
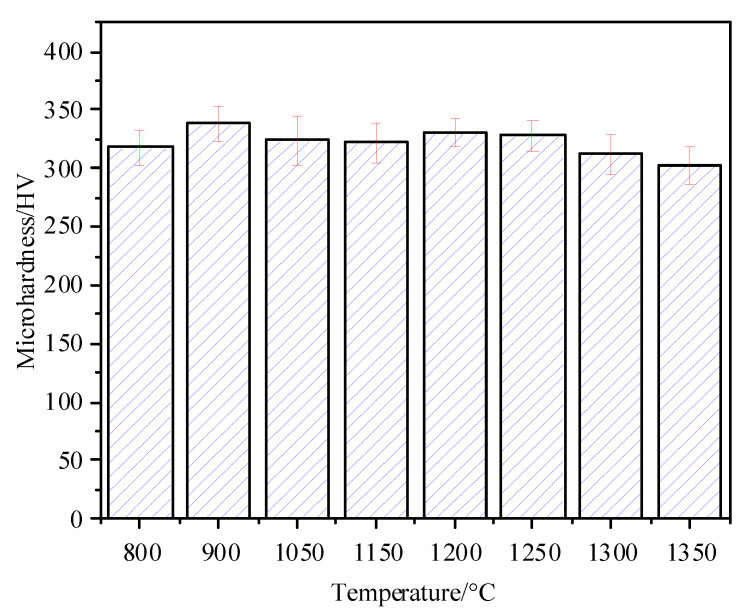
Microhardness values of simulated HAZ at different peak temperatures.

Figure 8 shows the change of Vickers hardness of the simulated HAZ. It indicates that the Vickers hardness of the simulated HAZ first increased and then trended to balance with the increase in peak temperature. The change in hardness at different peak temperatures is caused by the change in microstructure and grain size. The microhardness of the ICHAZ is 317.78 ± 15.05 HV. ICHAZ has a relatively small hardness; the temperature of the ICHAZ is located in the two-phase zone and the original microstructure is partially austenitized. The non-austenitizing microstructure undergoes recovery and recrystallization, accompanied by dislocation disappearance or polygonization, resulting in a ferrite microstructure with a lower hardness. The hardness value of the simulated FGHAZ is the largest, ranging from 323.97 ± 21.19 HV to 337.97 ± 21.19 HV, mainly because its structure is fine and it has a uniform hard phase structure, such as LM LB and a small amount of GB.

For the simulated CGHAZ, the Vickers hardness first increases and then decreases with the increase in peak temperature, but the change is not significant. When the peak temperature increases from 1150 °C to 1200 °C, the microhardness of CGHAZ increases by 8.91 HV. The main reason is that the content of LM with a higher hardness increases, and the content of LB and GB with relatively low hardness decreases. Although the microstructure has grown, it is still relatively small, so the hardness is increased. When the peak temperature exceeds 1200 °C, the Vickers hardness decreases with the increase in peak temperature. When the peak temperature reaches 1350 °C, the hardness is the lowest (302.59 ± 15.94HV). The reason for this is that the peak temperature is too high, and the microstructure is too coarse, which causes the microhardness to decrease. At the same time, with the increase in peak temperature, the content of LM with a higher hardness is still increasing, while the content of LB and GB with relatively lower hardness is decreasing, thus increasing the hardness. Eventually, the hardness decreases, but the change is not significant.

### 3.3. Impact Toughness

**Figure 9:**
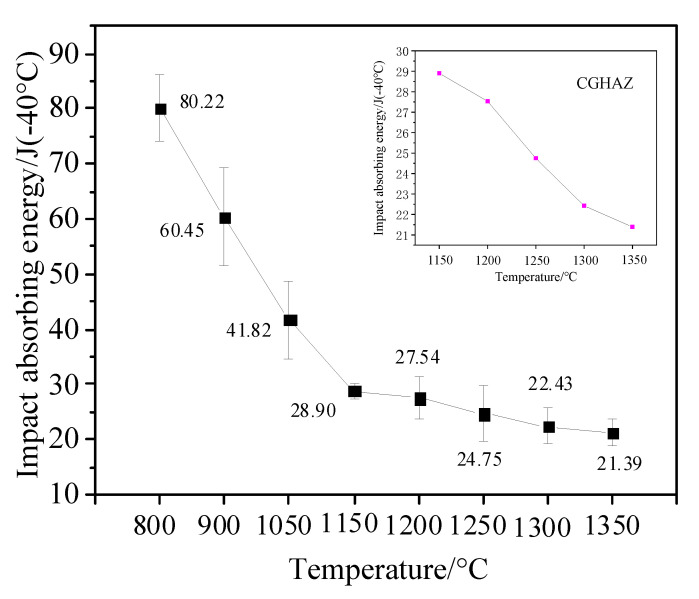
Impact toughness of simulated HAZ at different peak temperatures.

Affected by the welding thermal cycle, the microstructure of the HAZ continuously changes and has a specific gradient. The impact toughness of the welded joint has a complicated internal relationship with the microstructure and the welding process. Figure 9 shows the change of the −40 °C impact absorption energy of the experimental steel simulated HAZ with the peak temperature. The impact toughness of the simulated HAZ first sharply decreases, from 80.22 J to 28.9 J at a peak temperature of 800 °C–1150 °C, and then gradually decreases to 21.39 J at the peak temperature of 1350 °C.

When the peak temperature is 800 °C, the microstructure of simulated ICHAZ has the maximum impact energy (80.22 J), which is mainly because the microstructure of the ICHAZ is fine bainite and ferrite with a good toughness. The combination of soft and hard dual-phase microstructure makes the impact toughness of ICHAZ the best. In addition, intragranular acicular ferrite (IAF) can hinder the initiation of cracks and improve the toughness [24].

The impact energies of the FGHAZ with peak temperatures of 900 °C and 1050 °C are 60.45 J and 41.82 J, respectively. The impact toughness of FGHAZ with the peak temperature of 900 °C is still good. Although the FGHAZ with the peak temperature of 900 °C has relatively poor toughness martensite, the impact toughness is still good due to the martensite lath being small and the content also being relatively small and the microstructure is mainly small LB. When the peak temperature increases from 900 °C to 1050 °C, the microstructure grows and the LM content increases, resulting in a decrease in impact toughness. When the peak temperature increases from 1050 °C to 1150 °C, the decreasing trend is very obvious. This reason for this is that when the peak temperature increases from 1050 °C to 1150 °C, and the microstructure changes from FGHAZ to CGHAZ, and the microstructure grows significantly. At this time, the microstructure is mainly coarse LM, the content of LB decreases, and there is coarse GB. A coarse GB microstructure is unfavorable to the low-temperature impact properties of materials.

For the CGHAZ, the impact absorption energies of the CGHAZ with peak temperatures of 1150 °C, 1200 °C, 1250 °C, 1300 °C, and 1350 °C were 28.9 J, 27.54 J, 24.75 J, 22.43 J, and 21.39 J, respectively. The impact energy of the CGHAZ decreased progressively with the increase of peak temperature, and the changing trend was not pronounced. With the increase in peak temperature, the content of LB with a good toughness in the CGHAZ microstructure decreased gradually, and the content of LM with a poor toughness increased gradually; however, the CGHAZ microstructure type had not changed. The microstructure gradually became coarse with the increase in peak temperature, resulting in a decrease of the impact toughness of the CGHAZ. In addition, there are reports that the homogenous grain size brought about a high toughness [25]. It is evident that the grain size of the HAZ is obviously coarse with a peak temperature, which is probably the primary cause for the variation of impact toughness.

**Figure 10:**
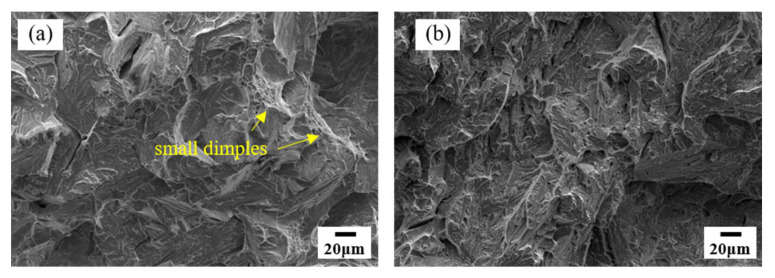
SEM micrographs of fracture surfaces of the simulated sample with peak temperature of (**a**) 1150 °C and (**b**) 1350 °C.

The CGHAZ has attracted a great deal of attention in the actual welding process, which is considered the main reason for the low-temperature impact toughness of the welding heat affected zone. Therefore, the fracture morphologies of simulated CGHAZ impact specimens were studied using SEM. Figure 10 shows the fracture surface with different peak temperatures. It can be seen that when the peak temperature is 1150 °C, there are small dimples (yellow arrow). The small dimples greatly promote the dissipated energy in the process of crack propagation and inhibit crack growth [5]. When the peak temperature is 1350 °C, the fracture surface is dissociation surface, and brittle fracture is easy to occur. For the coarse-grained region, this is one of the reasons why the toughness of 1150 °C is better than that of 1350 °C.

### 3.4. Crystallographic Characteristics

**Figure 11:**
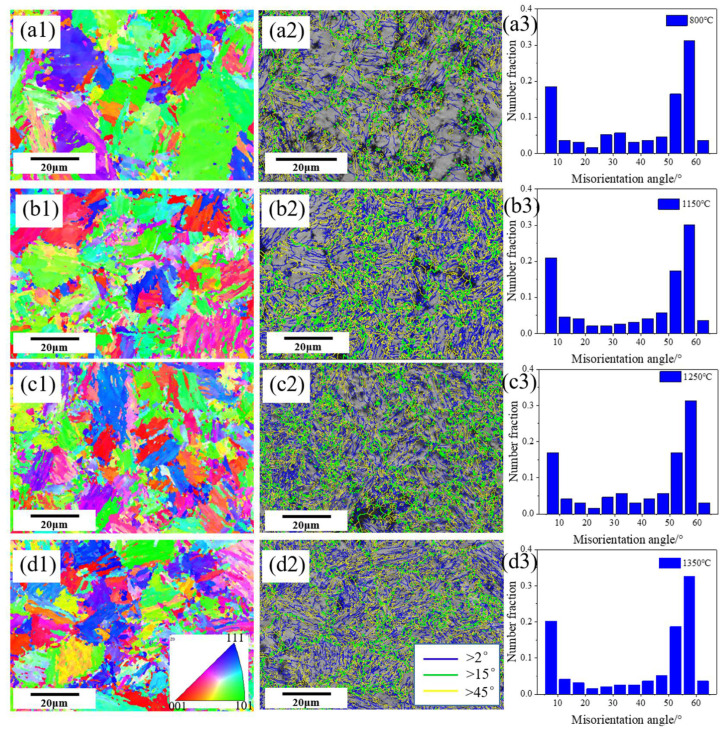
Crystallographic characteristics of the welded joint at different peak temperatures: (**a1**–**d1**) EBSD inverse pole figure (IPF); (**a2**–**d2**) grain boundary maps; (**a3**–**d3**) misorientation angle distribution histograms; (**a1**–**a3**) 800 °C; (**b1**–**b3**) 1150 °C; (**c1**–**c3**) 1250 °C; (**d1**–**d3**) 1350 °C.

**Figure 12:**
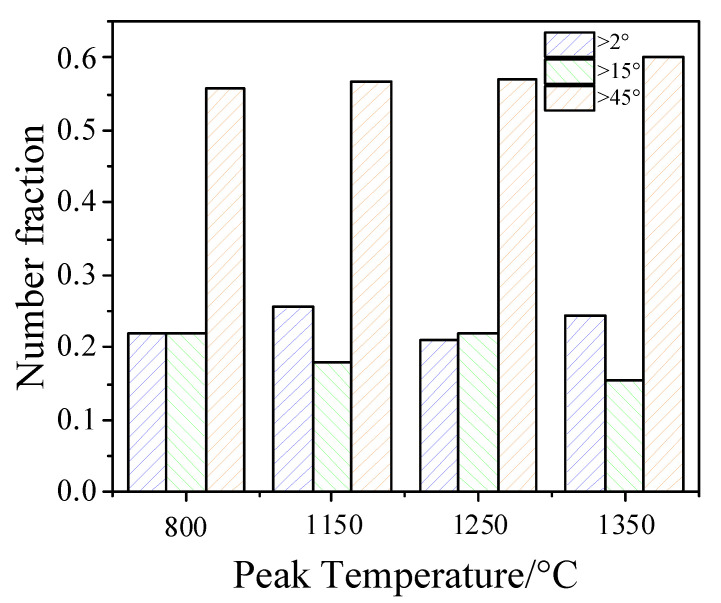
Misorientation angle distribution at different peak temperatures.

Some studies show that high-strength steels need more large-angle grain boundaries to prevent crack growth and improve toughness [26]. Large angle grain boundaries play an essential role in impact toughness. Large orientation differences can effectively avert crack propagation, thereby improving the impact toughness [27,28,29]. Therefore, using the EBSD method to characterize the crystallographic characteristics of its microstructure. Figure 11 shows the EBSD results with peak temperatures of 800 °C, 1150 °C, 1250 °C, and 1350 °C, respectively. Figure 11a1–d1 shows the orientation maps of the samples. Figure 11a2–d2 present the image quality maps with grain boundary misorientation distribution of the samples. The figure shows the grain boundary distributions, wherein the low misorientation boundaries of 2°–15° are depicted in blue lines. The high misorientation boundaries of 15°–45° and 45°–180° are depicted in green and yellow lines, respectively. Wang et al.’s [5] research shows that the grain boundary, which can hinder or deflect the crack propagation, is the grain boundary with an orientation difference greater than 45°. Therefore, the large-angle grain boundaries are divided into two ranges in this paper. Figure 11a3–d3 shows the histogram figures of the frequency of boundaries. Figure 11a3–d3 reflects the dominance of the low misorientation boundaries and the high misorientation boundaries. Figure 12 shows the statistical analysis of orientation differences in three ranges. When the peak temperature increases from 800 to 1350 °C, the change in the high angle grain boundary is not obvious. However, the impact toughness decreases by 58.83 J when the peak temperature increases from 800 to 1350 °C. This result is inconsistent with other scientific papers [29]. Therefore, we also observed the retained austenite in the microstructure.

**Figure 13:**
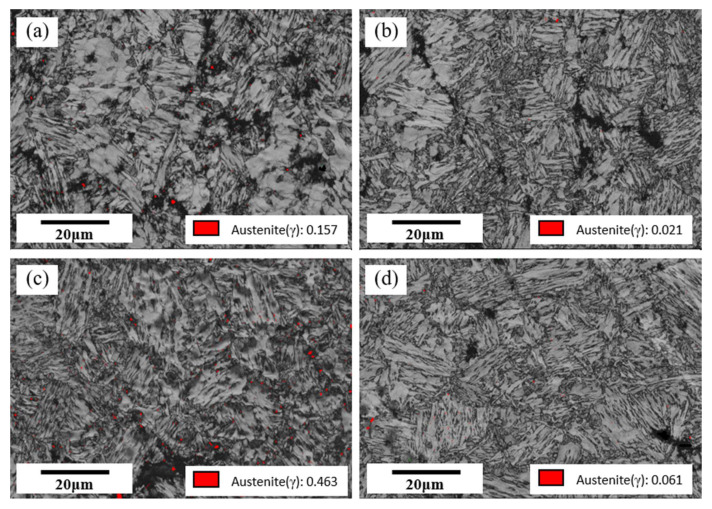
Image quality maps with austenite phase at different peak temperatures of (**a**) 800 °C; (**b**) 1150 °C; (**c**) 1250 °C; (**d**) 1350 °C.

Figure 13 shows image quality maps with the austenite phase of simulated samples at different peak temperatures. There is little retained austenite in the structure. With the increase in peak temperature, the change of retained austenite is not obvious. The results show that the content of the retained austenite is not the reason for the decrease in toughness.

## 4. Conclusions

The strengthening and toughening mechanisms of Q690 bridge steel were discussed by means of OM, SEM, EBSD, TEM, and mechanical properties tests. The conclusions are as follows:

(1) The microstructure of ICHAZ (800 °C peak temperature) consists of fine B and F. The microstructure of FGHAZ (900 °C and 1050 °C peak temperature) and CGHAZ (1150–1350 °C peak temperature) was composed of fine LB + GB + LM, but the microstructure of FGHAZ was finer.
(2) Microhardness: FGHAZ > CGHAZ > ICHAZ. The microhardness of the ICHAZ is the smallest, the microhardness of the FGHAZ is the largest, and the microhardness of CGHAZ is slightly less than that of the FGHAZ. With the increase of peak temperature, the microhardness of the CGHAZ first increases and then decreases.
(3) The −40 °C impact energy of HAZ decreases with the increase in peak temperature. When the peak temperature is between 800 °C and 1150 °C, the microstructure changes, and the impact energy decreases significantly. When the peak temperature is between 1150 °C and 1350 °C, the microstructure type remains unchanged, and the impact absorption energy decreases slowly. For the CGHAZ, when the peak temperature is 1200 °C, it has a high hardness and high toughness.
(4) Reasonable control of peak temperature is the key to prevent grain coarsening by studying the HAZ of Q690 steel. For CGHAZ, the peak temperature should be lower than 1200 °C to avoid a decrease in mechanical properties.
(5) The peak temperature increases from 800 °C to 1150 °C, the impact toughness decreases significantly, but the change in the high angle grain boundary is not apparent. The difference in microstructure and the original austenite grain size is the main reason for the difference in impact toughness.

## Figures and Tables

**Table 1:** Chemical composition of the experimental steel (wt%).

C	Si	Mn	Cr	Ni	Cu	Mo	Nb	V	Ti	B	Fe	CE
<0.10	0.20	1.30	0.50	1.00	0.30	0.70	0.03	0.08	0.012	0.0015	Bal	0.66

## Data Availability

The data is avaiable on the request to the corresponding author.
